# Editorial: Kinesiophobia – psychological aspects of physical activity in breast cancer patients

**DOI:** 10.3389/fpsyg.2024.1380019

**Published:** 2024-02-19

**Authors:** Ewa Malchrowicz-Mośko, Zbigniew Waśkiewicz, Arkaitz Castañeda-Babarro, Patxi León-Guereño

**Affiliations:** ^1^Department of Sports Tourism, Faculty of Physical Culture Sciences, Poznan University of Physical Education, Poznan, Poland; ^2^Health, Physical Activity and Sports Science Laboratory (HealthPASS), Department of Physical Activity and Sports, Faculty of Education and Sports, University of Deusto, Bilbao, Spain; ^3^Department of Physical Activity and Sports, Faculty of Education and Sports, University of Deusto, Donostia-San Sebastián, Spain

**Keywords:** kinesiophobia, breast cancer, physical activity, psychology, wellbeing, barriers

Breast cancer is one of the most serious diseases and with the highest incidence affecting women worldwide. Investigations have shown that physical activity and sports are important elements in both, the prevention of breast cancer and its recurrence after cancer treatment. Bibliography shows that physical activity can favors patients' physical and mental health and improve treatment outcomes even during oncological treatment. Even though oncological patients were previously advised to lead a quiet and sedentary life, recent research has shown that physical activity should play a prominent role in treating and preventing cancer recurrence.

Despite this evidence, the inclusion of physical activity is sometimes discussed between patients and doctors following a cancer diagnosis. There are various types of barriers to physical activity (social, religious, cultural, economic, and psychological), and the current pandemic is adding new difficulties to practicing physical activity, including fear of infection. Additionally, many patients suffer from kinesiophobia or fear of movement due to the expected pain or excessive fatigue. Therefore, many patients remain unaware of the importance of incorporating physical activity as part of their cancer treatment. Indeed, initiatives promoting an active lifestyle among oncological patients (like collective runs, and Nordic walk meetings) sometimes do not reach elderly patients, people living far away from oncology centers, and patients with low education levels and/or low socioeconomic status. Women are particularly affected by these barriers, therefore breast cancer patients are even less likely to engage in regular physical activity after being diagnosed.

Thus, it is essential to reflect on the problem of kinesiophobia among patients suffering from breast cancer and to discuss effective methods of managing patients' attitudes toward physical exercise, especially during the current pandemic. Therefore the main objective of this Research Topic is gather manuscripts related to physical activity of patients diagnosed with breast cancer and their relationship with physical activity and an active lifestyle during and after the disease.

This Research Topic *Kinesiophobia – psychological aspects of physical activity in breast cancer patients* of the *Frontiers in Oncology*, includes articles related to the measurement and validation of quality-of-life of breast cancer inpatients, the level of fear or kinesiophobia of Breast Cancer patients undergoing surgical treatment, the analysis of the influence of breast cancer treatment on survivor women initiating or related to their physical activity adherence, or variables like the influence of psychosocial and behavioral factors on Cancer Related Cognitive Impairment manifestations, have been analyzed.

Among the studies related to physical activity and breast cancer, Malchrowicz-Mośko et al. examined the fear of movement among patients with breast cancer in Poland, and the results of study published indicated the occurrence of the phenomenon of kinesiophobia. It has been observed that there is a relationship between the level of kinesiophobia and age—the older the women, the higher the level of kinesiophobia. The highest level of kinesiophobia concerns older breast cancer patients who are also diagnosed with overweight, obesity, insulin resistance, type II diabetes and osteoporosis (often leading to osteoporotic fractures). Data from the literature indicate that the above-mentioned chronic diseases are one of the factors contributing to the development of breast cancer. At the same time, systemic treatment undertaken after diagnosis (hormone therapy) may intensify the progression of these diseases, resulting in increased body weight, decreased bone density and decreased insulin sensitivity (Andò et al., 2019).

Half of the women surveyed declared that they were not physically active during cancer treatment and did not meet the recommendations of the World Health Organization (WHO, 2020), which emphasizes that cancer patients should perform the same weekly dose of physical activity as healthy people. Similar research (Malchrowicz-Mośko, 2022) has shown that the phenomenon of kinesiophobia persists a year after the end of hospital treatment for breast cancer, and every third woman is not physically active at that time. High levels of kinesiophobia correlated with low levels of physical activity, and women with obesity and diabetes declared higher levels of kinesiophobia 1 year after treatment than women without comorbidities. Moreover, lifestyle before diagnosis affects the level of kinesiophobia after treatment—women who were physically inactive before the diagnosis of the disease declared a higher level of fear of movement than women who were previously active. Unfortunately, patients often do not associate their lifestyle as a potential cause of the disease. Lack of physical activity, unlike genes or environment, is modifiable and may protect against disease recurrence and the occurrence of sarcopenia and osteosarcopenia. It is estimated that 25% of cancer cases worldwide are caused by overweight and a sedentary lifestyle (McTiernan, 2008).

Unfortunately, there is a common belief among patients that they need to avoid physical activity due to unjustified fear of pain, fatigue or cancer metastasis. Considering that systemic treatment of breast cancer lasts up to 15 years (hormone therapy)—it is important to eliminate kinesiophobia at every stage of cancer treatment. Therefore, the undertaken research indicated the occurrence of the phenomenon of kinesiophobia and the insufficient participation of women after breast cancer in physical activity using the Tampa Scale of Kinesiophobia measuring the level of kinesiophobia (Rozmiarek et al., 2022). Moreover, not all patients join breast cancer patient organizations which promote physical activity and healthy lifestyle, so every woman in the hospital or oncology center should be educated in this area, especially the elderly, those with comorbidities and living away from health centers. Their sense of agency and influence on the course of the disease should be strengthened.

On the other hand, Li et al. developed and validated the Breast Cancer Scale QLICP-BR V2.0. Based on Classical Test Theory and Generalizability Theory in a Chinese population. This quality-of-life related tool for cancer patients (QLICP-BR V2.0), showed a reasonable degrees of validity, reliability, and responsiveness according to classical test and the generalizability theory, and the number of items in the scale was considered appropriate, therefore creating a practical and useful survey. In another study, Sequeira et al., in the Portuguese context, carried out a qualitative research, in which the objective was to explore the perspectives of women survivors of breast cancer regarding regular physical activity. Breast cancer patients' physical activity needs and preferences were considered important to increase their adherence, and the main findings of this research suggested that emotional factors and beliefs of patients should be considered when planning a successful intervention for this population. Finally, West et al., analyzed the impact of psychosocial and behavioral factors on Cancer Related Cognitive Impairment manifestations in the Italian context. In total 233 women, of which 106 were breast cancer patients and 127 were age-matched controls without oncological diagnosis, completed the questionnaire. Variables like cognitive functionality, sociodemographic characteristics, clinical information, psychosocial and behavioral factors (cognitive reserve, sleep quality, dietary habits, physical activity) were analyzed, showing statistically significant differences between sample groups.

In conclusion, the studies collected in this Research Topic shed some light regarded to breast cancer survivors and their physical activity behaviors, trends and preferences, ultimately, allowing us to better understand the relationship that patients who have suffered from breast cancer have with physical activity, and thus be able to improve and adapt these practices to their needs, increasing their adherence and ultimately their quality of life and health.

## Author contributions

EM-M: Conceptualization, Supervision, Writing—original draft, Writing—review & editing. ZW: Supervision, Writing—review & editing. AC-B: Writing—review & editing, Supervision. PL-G: Methodology, Writing—original draft, Writing—review & editing.
